# Preferentially Quantized Linker DNA Lengths in *Saccharomyces cerevisiae*

**DOI:** 10.1371/journal.pcbi.1000175

**Published:** 2008-09-12

**Authors:** Ji-Ping Wang, Yvonne Fondufe-Mittendorf, Liqun Xi, Guei-Feng Tsai, Eran Segal, Jonathan Widom

**Affiliations:** 1 Department of Statistics, Northwestern University, Evanston, Illinois, United States of America; 2 Department of Biochemistry, Molecular Biology and Cell Biology, Northwestern University, Evanston, Illinois, United States of America; 3 Department of Chemistry, Northwestern University, Evanston, Illinois, United States of America; 4 Center for Drug Evaluation, Taipei, Taiwan, Republic of China; 5 Department of Computer Science and Applied Mathematics, Weizmann Institute of Science, Rehovot, Israel; Washington University, United States of America

## Abstract

The exact lengths of linker DNAs connecting adjacent nucleosomes specify the intrinsic three-dimensional structures of eukaryotic chromatin fibers. Some studies suggest that linker DNA lengths preferentially occur at certain quantized values, differing one from another by integral multiples of the DNA helical repeat, ∼10 bp; however, studies in the literature are inconsistent. Here, we investigate linker DNA length distributions in the yeast *Saccharomyces cerevisiae* genome, using two novel methods: a Fourier analysis of genomic dinucleotide periodicities adjacent to experimentally mapped nucleosomes and a duration hidden Markov model applied to experimentally defined dinucleosomes. Both methods reveal that linker DNA lengths in yeast are preferentially periodic at the DNA helical repeat (∼10 bp), obeying the forms 10*n*+5 bp (integer *n*). This 10 bp periodicity implies an ordered superhelical intrinsic structure for the average chromatin fiber in yeast.

## Author Summary

Eukaryotic genomic DNA exists as chromatin, with the DNA wrapped locally into a repeating array of protein–DNA complexes (“nucleosomes”) separated by short stretches of unwrapped “linker” DNA. Nucleosome arrays further compact into ∼30-nm-wide higher-order chromatin structures. Despite decades of work, there remains no agreement about the structure of the 30 nm fiber, or even if the structure is ordered or random. The helical symmetry of DNA couples the one-dimensional distribution of nucleosomes along the DNA to an intrinsic three-dimensional structure for the chromatin fiber. Random linker length distributions imply random three-dimensional intrinsic fiber structures, whereas different possible nonrandom length distributions imply different ordered structures. Here we use two independent computational methods, with two independent kinds of experimental data, to experimentally define the probability distribution of linker DNA lengths in yeast. Both methods agree that linker DNA lengths in yeast come in a set of preferentially quantized lengths that differ one from another by ∼10 bp, the DNA helical repeat, with a preferred phase offset of 5 bp. The preferential quantization of lengths implies that the intrinsic three-dimensional structure for the average chromatin fiber is ordered, not random. The 5 bp offset implies a particular geometry for this intrinsic structure.

## Introduction

Eukaryotic genomic DNA exists in vivo as a hierarchically compacted protein-DNA complex called chromatin [1]. In the first level of compaction, 147 bp lengths of DNA are wrapped in 1 3/4 superhelical turns around protein spools, forming nucleosomes [2]. Consecutive nucleosomes are separated by short stretches of unwrapped “linker” DNA. Most chromatin in vivo is further folded into shorter, wider fibers, ∼30 nm in diameter. Despite much effort, the structure of the 30 nm fiber remains unresolved [3],[4].

Here we report that an analysis of the relative locations of nucleosomes along the DNA sheds new light on chromatin fiber structure. The connection arises from the helical symmetry of DNA itself [5]–[8]. Each base pair increase in separation between two consecutive nucleosomes moves them apart by 0.34 nm along the DNA - a potentially minor change relative to the 30 nm fiber's width. However, because of the 10.2–10.5 bp per turn helical symmetry of DNA, this 0.34 nm translation is coupled to a ∼35° rotation about the DNA helix axis, rotating the second nucleosome to an entirely different location in space, creating an entirely different intrinsic chromatin structure.

In vivo, attractive nucleosome-nucleosome interactions [9],[10] might overwhelm this intrinsic structure for the chromatin fiber, and impose a particular folded structure that is independent of exact linker DNA lengths. In that case, changes in the fiber's intrinsic structure would be manifested instead as changes in the folded fiber's stability [8]. Because of the high torsional stiffness of DNA and the short lengths of linker DNAs, such changes in stability would be of great energetic significance.

While steps of one or several bp profoundly alter the intrinsic fiber structure, steps of 10–11 *n* bp (integer *n*) do not: instead, the next nucleosome rotates *n* complete turns around the DNA helix axis, ending up rotationally near where it began, but translated along the DNA by ∼3.4–3.7 *n* nm. If linker DNA lengths varied randomly about an average value, the resulting intrinsic chromatin structure would be a random flight chain. But if linker DNA segments instead were equal in length modulo the DNA helical repeat, this would define an intrinsically ordered (but possibly irregular) superhelical structure for the chromatin fiber, with the detailed intrinsic structure highly depending on the phase offset *d*_0_ (integer) for linker DNAs of length 10*n*+*d*_0_ bp.

There are many hints in the literature for a ∼10 bp-periodicity in lengths of linker DNAs [5]–[8], [11]–[13]; however, the results are inconsistent. An early analysis of oligonucleosome DNA lengths suggested that linker DNAs in the yeast *S. cerevisiae* preferentially occur in lengths of 10*n*+5 bp, while those in human HeLa and chicken erythrocyte cells have no periodicity [5]. Analogous studies on rat liver chromatin first did not [14], but later did [6], reveal periodic linker DNA lengths, again of the form 10*n*+5 bp. Later genome-wide correlation analyses of AA and TT dinucleotides (which favor particular locations within the nucleosome [15],[16]) similarly yielded variable results, suggesting preferences of the form 10*n*+5 [11] or ∼10.6*n*+8 bp for yeast [12], ∼10.6*n*+8 for *Caenorhabditis elegans* and *Drosophila melanogaster*, [12], and ∼10*n*+8 for human k562 cells [13].

These conflicting conclusions of existing studies motivated us to develop two new independent computational methods and new experimental data, to define the probability distribution of linker DNA lengths in yeast. Our results from both approaches show that linker DNA lengths in yeast are indeed preferentially periodic, implying that the yeast genome encodes an intrinsically ordered three-dimensional structure for its average chromatin fiber.

## Results

### Fourier Analysis of Extended Dinucleotide Frequency

**Figure 1:**
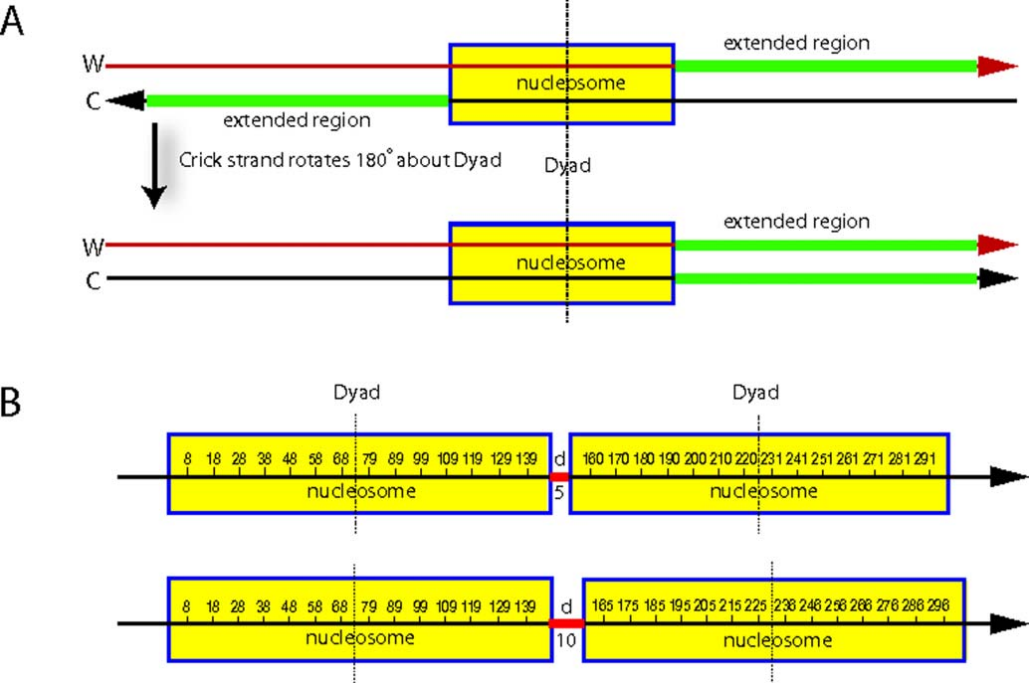
A diagram for extended dinucleotide frequency analysis. (A) How the extended sequences are obtained on the genome. DNA sequence from experimentally mapped nucleosomes is extended in the 3′ direction on both strands. AA/TT/TA signals, when combined, are center symmetric, hence information from the 5′-extended sequence is implicitly included. (B) Preferred locations of AA/TT/TA signals over two consecutive nucleosomes, for linker DNA lengths of *d* = 5 bp (top) and 10 bp (bottom), respectively.

A well-known characteristic of nucleosome DNA sequences is the ∼10 bp periodicity of key dinucleotide motifs, particularly AA, TT, TA, and GC. AA/TT/TA steps occur in phase with each other, and out of phase with GC [16]–[18]. These facts allow one to test for genomically encoded preferences in linker DNA lengths. Consider a set of experimentally mapped nucleosome sequences **S** = {*s*_1_, …, *s_i_*, … *s_I_*}, strictly aligned at their dyad (2-fold rotational symmetry) axes. Extend each aligned sequence in both directions along the genome by roughly one nucleosome length (Fig 1A, note: positions from 1 to 147 stand for the nucleosome region). Occurrences of AA/TT/TA motifs as a function of position in the flanking regions of **S** would then exhibit collective patterns that are determined by the distribution of linker DNA lengths around the central (mapped) nucleosomes. If the central nucleosomes were perfectly aligned, and linker lengths were a constant, *d*_0_, then the nucleosomes in the up-/downstream region of **S** would also be strictly aligned. A significant periodic signal from AA/TT/TA motifs would then occur at up-/downstream positions dependent upon *d*_0_ (Figure 1B). If instead the *i*th linker length downstream of sequence *s_i_*, *l_i_*, equals 10*n_i_*+*d*_0_ for some integer *n_i_* and a fixed *d*_0_ (0≤*d*_0_<10), then the nucleosomes immediately downstream of **S** would not be strictly aligned, but would instead be offset by a multiple of 10 bp relative to each other. In this case, the ∼10 bp periodicity of dinucleotide motif signals would be roughly maintained in the extended regions, but more weakly, since end regions of the adjacent nucleosomal DNA in some sequences would be partially aligned over linker DNA in other sequences. Alternatively, if linker DNA lengths were random, these dinucleotide motifs would lack any significant periodicity in the extended regions.

**Figure 2:**
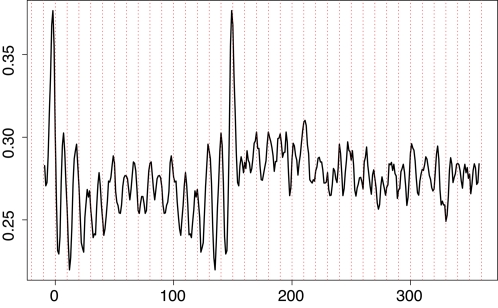
AA/TT/TA dinucleotide frequency in extended nucleosome sequence alignment. With a combined encoding of AA/TT/TA dinucleotides, the upstream sequences are center symmetric with the downstream; only the downstream regions are plotted. Positions 1–147 corresponds to the original center alignment of the mapped nucleosome cores, while 148 and above are the extended regions.

We used this approach to test for intrinsically encoded linker DNA length preferences in the yeast genomes. Our in vivo yeast nucleosome sequence collection (filtered for nonredundant sequences of length 142–152 bp) contains 296 sequences. We focus the analysis on the AA/TT/TA signal because this is the most strongly periodic in aligned nucleosome sequences [16]. Alignment of these sequences (Figure 2) reveal several striking features. Sharp signals at positions −1 and 149, and systematic differences in average AA/TT/TA frequency between the original mapped nucleosomes and the extended regions, may reflect sequence preferences of the micrococcal nuclease [19] which is used to biochemically isolate the nucleosomes, or may reflect sequence preferences intrinsic to nucleosomes and linker regions [20].

Most importantly, the plot reveals hints of a ∼10 bp periodicity in the extended regions, implying that the yeast genomes intrinsically encode preferentially quantized linker DNA lengths of the form ∼10*n*+*d*_0_. The value of *d*_0_ can be deduced from the positions of the AA/TT/TA peaks in the extended region. Assume the AA/TT/TA signal appears periodically at positions 8, 18, …79, … 139 within a nucleosome region [15],[16]. If the linker length is 5 bp (or more generally 10*n*+5), then the expected positions of AA/TT/TA peaks (as indexed in Fig. 1B) in the downstream nucleosome region would be 160, 170,…, 231,… 291 (or these indices+10*n* if the linker length is 10*n*+5). In accord with this analysis, the AA/TT/TA peaks in Figure 2 are roughly positioned at 10's or 1's in the downstream region. Therefore we conclude that the preferential linker length value in the yeast data obeys the rule 10*n*+5(*d*_0_ = 5).

**Figure 3:**
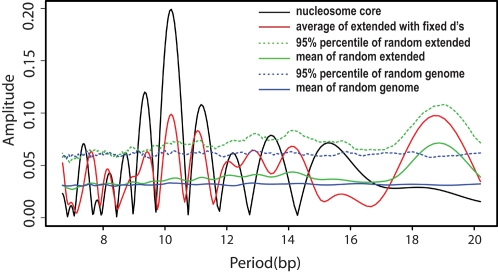
Fourier amplitude spectra of TT/AA/TA signals in nucleosome core/extended regions, compared to randomly shifted samples and random genomic samples.

To test the significance of the observed 10 bp periodicity, we first calculated the Fourier transform of the AA/TT/TA signal in the extended region from position 147+*d* to 147+*d*+180 (Figure 2) for a given *d*. We chose *d* to be greater than 10 to avoid the sharp peaks observed at the boundaries of mapped nucleosome sequences (at positions −1 and 149 in Figure 2) that likely owe to the micrococcal nuclease enzyme specificity. We then varied *d* from 11 to 20. The amplitude spectrum (square root of spectral power) averaged over all *d*'s is plotted as a function of period in Figure 3 (red curve). As a control, we constructed 500 randomly shifted samples of the extended regions by choosing a random *d_i_* value between 11 and 20 for each sequence *s_i_*, *i* = 1, … *I* (see details in Methods). As another control, we obtained 500 random genomic samples, each sample containing the same number of sequences of the same length (180 bp) randomly selected from the genome. The mean and 95% percentile of the Fourier transform amplitude at each periodicity value derived from these two sets of control samples are also plotted in Figure 3.

A significant peak at the 5% level (i.e., where the average amplitude from the extended samples with fixed *d*'s (red curve) exceeds the 95% percentile line from the randomly shifted samples (green dashed line) or random genomic samples (blue dashed line)) is observed at period ∼10.2 bp. Because multiple peaks exist around 10 bp periodicity, we use the total power corresponding to period between 9–11 bp as the test statistic. The total power at 9–11 bp averaged over different *d*'s was compared with that of the random shifted and random genomic samples. The resulting *p*-values are 0.008 and 0 respectively, refuting the hypothesis that linker DNA lengths within any 10 bp range are uniformly preferred in the genome. Instead, these significant ∼10 bp periodicities are consistent with the hypothesis that linker lengths in the yeast genome prefer values of the form ∼10*n*+*d*_0_, for some constant *d*_0_.

Information about preferred values for *d*_0_ is contained in the phase of the corresponding ∼10 bp periodicity peak in the Fourier transform. In Table 1 we present the location of the Fourier transform amplitude peak (T*) around 10 bp periodicity, and the corresponding phase angle in radians, for all *d*'s. If the experimentally obtained nucleosome sequences were perfectly aligned, and if linker DNA lengths were genuinely 10*n*+*d*_0_ for a constant *d*_0_, then shifting the downstream region leftward by *d*_0_ bp will synchronize the extended region's AA/TT/TA motif signal with that in the original mapped nucleosome region. For example, suppose the true linker length is 15 bp (i.e., 10*n*+*d*_0_ with *n* = 1 and *d*_0_ = 5). As indexed in Figure 1, the downstream nucleosome AA/TT/TA peaks will be positioned at 170, 180, … 301. By shifting the downstream region leftward by 5 bp, we will extract the region comprising basepairs 153 ( = 147+5+1) and above. The AA/TT/TA peaks in the extracted region now are positioned at basepairs 18, 28,…149 (relative to the first basepair of the extracted region). We hence expect the phase of Fourier transform at period ∼10 bp from this extracted region to be close to that from the original mapped nucleosome sequences, which had AA/TT/TA peaks positioned at 8, 18,…139 relative to their own first basepairs. Consistent with this analysis, the phase of the AA/TT/TA signal when *d* = 15 (i.e., when the extended region begins on the 16th basepair after the end of the mapped nucleosomes) is 2.56 in radians, closest among all *d*'s to 2.48, the phase from the original mapped nucleosomes. Based on this criterion therefore, we conclude the optimal *d*_0_ is 5 bp.

**Table 1:** Fourier analysis of AA/TT/TA motif signal in extended region.

	Mono	Extended, *d*									
		11	12	13	14	15	16	17	18	19	20
Amplitude (A*)	0.208	0.098	0.103	0.101	0.100	0.101	0.100	0.099	0.096	0.098	0.098
Period (T*)	10.20	10.20	10.20	10.20	10.20	10.20	10.20	10.20	10.20	10.20	10.20
Phase (*φ_d_*)	**2.48**	0.04	0.64	1.31	1.91	**2.56**	−3.12	−2.49	−1.85	−1.32	−0.68

*T* ^*^ is the period that corresponds to the largest amplitude peak between 9 and 11 bp; *φ_d_* the corresponding phase angle in radians (−*π*≤*φ_d_*≤π); and *A* ^*^ is the amplitude at *T* ^*^, for extended regions beginning *d* bp beyond the aligned experimentally mapped nucleosomes *φ*.

This phase analysis for detecting the preferred quantized linker DNA lengths (i.e., the preferred *d*_0_) assumes that the AA/TT/TA motif maintains the same periodicity in the extended region as in the mapped nucleosomes. This is true: the periodicity having maximum Fourier amplitude (T*) equals 10.20 bp for both the mapped nucleosomes and the extended regions (Figure 3 and Table 1). Hence this analysis implies that linker DNA lengths in yeast are preferentially quantized, with the form ∼10.2*n*+5 bp. The amplitude in the extended region however is much lower than in the original core region. This may suggest that that the linker length distribution is not strictly quantized at (odd) multiples of 5's, but rather in a form possessing non-degenerate peaks centered around (odd) multiples of 5's.

### Duration Hidden Markov Model (DHMM)

To test the conclusions of the Fourier analysis described above, and to better define the preferred phase offsets *d*_0_, we developed a duration hidden Markov model (DHMM, [21]) and used it to analyze a new collection of DNA sequences of dinucleosomes from yeast which we isolated for this purpose. Dinucleosomes are two nucleosomes connected by their linker DNA. The DHMM models the dinucleosomes as an oscillating series of two “hidden” states: a fixed-length (147 bp) nucleosome and a variable length linker DNA. A technical detail is that, as isolated biochemically, dinucleosomes may come with additional short partial linker DNA segments at either end, or alternatively, may be over-digested so as to have an incomplete nucleosome at either end. We generalize our DHMM to allow for this possibility. The algorithm predicts the positioning of two nucleosomes (complete or partial) in each sequence, and then uses the predicted results to update parameters in the model that describe the length and sequence preferences of the linker DNA. In particular, as the algorithm proceeds iteratively, the linker length distribution is updated using the kernel smoothing method (see details in Methods).

We isolated and fully sequenced 335 non-redundant dinucleosomes from yeast, with lengths ranging from 280 to 351 bp. Some of the dinucleosome sequences were shorter than 2*147 bp, meaning that they have been over-digested on at least one of their two ends. For such sequences the optimal path is more difficult to predict because of loss of information in either end. Hence we restricted our analysis to 214 sequences whose lengths are ≥300 bp.

**Figure 4:**
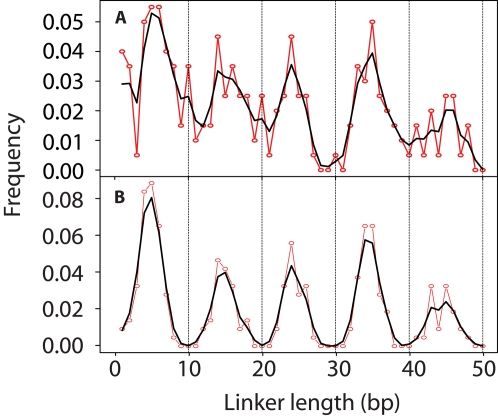
Linker length distribution predicted under kernel smoothing (A) and mixture model (B). The red curve is the raw frequency and the black is the smoothed curve using a 0.75 bp bandwidth Gaussian kernel and shown for convenience as a continuous curve.

At convergence of the model, the results (Figure 4A) confirm the results from the independent Fourier analysis of extended mononucleosome sequences (Figure 2 and Table 1, above): the linker length distribution function *F_L_*(*d*) obeys the rule 10*n*+*d*_0_ with the phase offset *d*_0_ = 5 bp, such that the most probable linker lengths (in the kernel-smoothed distribution) are around 5, 15, 25, 35, and 45 bp.

The noise reflected in Figure 4A comprises two chief components: individual major peaks can be slightly offset from 5's; also small peaks arise at seemingly random positions. This variability in the estimated density curve is not surprising, since we are estimating a distribution in an infinite-dimensional space. To reduce the dimensionality of the problem, we next consider a parametric approach in which we impose a periodicity on the linker length distribution function, *F_L_*(*d*), while allowing variability around each period. Such a distribution can be characterized by a mixture of Gaussian distributions with means that are equally spaced by 10 bp, and a common unknown variance (see Methods). The algorithm proceeds in the same way as the kernel smoothing method above, except that the linker length distribution is estimated using an EM algorithm.

The results at convergence of the model (Figure 4B) imply that, if the linker lengths do indeed prefer the form specified in this model, then the optimal *d*_0_ value is ∼5 bp. The estimate of the common standard deviation of the Gaussian components was 1.43, indicating a modest uncertainty of the linker length distribution around the quantized values. We further generalized the linker length model by treating the period as an unknown parameter and assuming heterogeneity in the variance of Gaussian components. The resulting maximum likelihood estimator of the period is 9.8 bp and the linker length distributions closely resemble those of Figure 4B (results not shown). Taken together, these results confirm the results of the Fourier analysis and of the DHMM kernel smoothing analyses. All of these analyses imply that linker DNA lengths in yeast obey the form 10*n*+*d*_0_ with *d*_0_ equal to ∼5 bp.

### Additional Tests of the DHMM Analyses

One possible concern in the DHMM analyses is whether the ∼10 bp periodicity in the linker length distribution could somehow arise from the model itself, especially given the ∼10 bp periodicity of motif signals inherent in the nucleosomes. Two simulation studies tested and disproved this possibility. One test simulated random sequences based on a product multinomial model with base composition and length distribution identical to that in the true dinucleosome sequences; the second test shuffled the natural dinucleosome sequences while keeping the dinucleotide frequency fixed within each sequence. The DHMM-kernel procedure was followed exactly as before. In both simulations, the resulting linker length distribution varied between trials, and the ∼10 bp periodicity disappeared in general (Figure S1). The DHMM-mixture method imposes the 10 bp periodicity on the linker length, but the peak positions often moved little away from their initial values of *μ* as the algorithm proceeded - presumably because, unlike for the real sequences, the randomized sequences lack signals that spur the movement of *μ* in the true nucleosome sequences. Thus, the real data are distinguished from the random data in both versions of the DHMM. We conclude that the linker length patterns deduced by these analyses reflect true signals of nucleosome organization present in the dinucleosome sequences.

**Figure 5:**
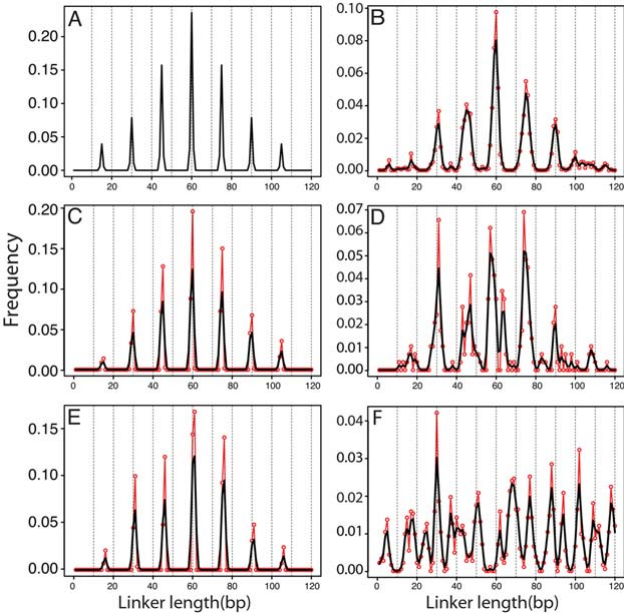
Linker DNA length distribution predicted for simulated dinucleosome sequences with periodic linker length (A–E) and uniform linker length (F). Linker DNA length distribution predicted for simulated dinucleosome sequences with 15 bp periodic linker length (A–E) and uniform linker length (F). (A) True linker length distribution used for simulation of dinucleosome sequences. (B, C) Recovered linker length distribution under the kernel and mixture methods, respectively, for 2,000 simulated dinucleosome sequences. (D, E) Corresponding results for a subset of the simulated dinucleosomes comprising only 300 sequences. (F) Recovered linker lengths using the kernel method for 2000 simulated dinucleosome sequences under the same model as in (B )but with a uniform linker length distribution on [1,…120].

To evaluate the robustness of these DHMM analyses to over- or under-digestion of the biochemically isolated nucleosomes and dinucleosomes, we carried out a simulation of the entire combined experiment. We simulated 2000 nucleosome sequences based on the experimentally obtained yeast nucleosome profile (a heterogeneous Markov chain model). Both ends of each simulated nucleosome were subjected to a random truncation or addition to the 147 bp-long nucleosome core by up to 3 bp, creating a set of simulated yeast nucleosome sequences having lengths in the range 141–153 bp, slightly greater than the 142–152 bp range of lengths in the real nucleosome sequences. Similarly, we simulated 2000 dinucleosome sequences, each starting and/or ending with a (simulated) nucleosome that was subject to a random truncation or addition of up to 20 bp. The linker DNAs were simulated using the homogeneous Markov chain model obtained from the yeast dinucleosome data, while the true linker length distribution followed a periodic distribution with peaks at 15,30,…105 (Figure 5A). The length range of resulting dinucleosome sequences is ∼250–440 bp, which we again filtered to retain lengths greater than or equal to 300 bp. We followed the same center alignment and model training procedure as for the real data. The periodic linker length distribution was successfully recovered using both the kernel smoothing and mixture model approaches (Figure 5B and 5C). Similar results were obtained with a small subset (300) of the dinucleosome sequences (Figure 5D), where the superior performance of the mixture method on this smaller dataset is evident (Figure 5C vs. Figure 5B and 5E vs. Figure 5D). In another check, we simulated another 2000 dinucleosome sequences having a uniform distribution for the linker length on [1,…120]. The resulting predicted linker length distribution (Figure 5F) lacks significant periodicity; peaks formed randomly, and their positions varied from sample to sample.

**Figure 6:**
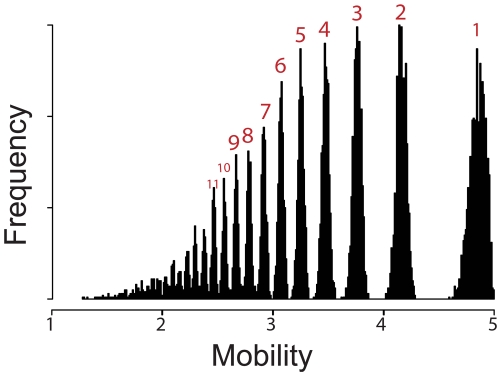
Simulated gel electrophoresis patterns under linker length distributions from the DHMM-mixture model. Frequency is plotted versus number of nucleosomes in each oligonucleosome fragment band, shown on a log scale (since mobility in a gel electrophoretic separation is proportional to the logarithm of DNA fragment lengths) with simulated electrophoresis from left to right.

Classic experimental measurement of the nucleosome repeat length provide several additional checks on the results from the DHMM analyses. Experiments using gel electrophoresis to analyze the DNAs that result from random partial nuclease digestion of chromatin routinely reveal ladder-like patterns of DNAs fragments, which reflect a repetition of a (relatively) discrete sized structural unit comprising a nucleosome plus one average linker DNA length. The length of DNA in this repeating unit is referred to as the nucleosome repeat length. Specifically, the nucleosome repeat length may be defined, and measured, as the average length difference in base pairs between DNA fragments containing *n*+1 and *n* nucleosomes. In one test of our analyses, we find that the average length of linker DNA for yeast predicted from the kernel smoothing method is 20.2 bp (21 bp from the mixture model), in good agreement with the experimental value of ∼18 bp for yeast [22], and in good agreement with subsequent studies suggesting that ∼20 bp may be a more-accurate value than ∼18 bp [3],[8]. As a second check on our analyses, we simulated the complete experimental measurement of nucleosome repeat length. We first simulated the chromatin fiber itself, given our linker length distribution function deduced from the DHMM analysis, then simulated the random partial nuclease digestion, and then finally simulated the gel electrophoresis analysis of the resulting DNA fragments. The simulated chromatin fibers comprised 50,000 nucleosomes with linker DNA lengths distributed as from the mixture model results, i.e., *μ* = (5,15,25,35,45), σ = 1.43 and η = (0.3271,0.1682,0.1636,0.2243,0.116) (*μ* was rounded to integers for convenience). The simulated nucleosome chain was then subjected to a simulated nuclease digestion. To mimic the partial nuclease digestion conditions used experimentally, each linker was subject to zero or one enzyme cut, at a random position and with probability proportional to its length, such that the resulting DNA fragments had a wide range of numbers of nucleosomes, with a mean of 5 nucleosomes. The simulated gel intensity profile (Figure 6) resembles those observed experimentally. Thus, the complex linker DNA length distributions deduced in our DHMM analyses are consistent with the experimental observations of ladder patterns in nuclease digestions of chromatin. Finally, as a third check on our analyses, we used these simulation-derived plots of frequency versus fragment size to recover an apparent nucleosome repeat length. The average repeat length based on 50 simulations was 168.5 bp with standard deviation 1.0, which accurately recovered the true theoretic repeat length for this modeled distribution, 147+21.3 = 168.3 bp.

We conclude from all of these tests that the complex linker DNA length distribution functions deduced with our DHMM analyses represent true features in the dinucleosome DNA sequences, and that they are compatible with available experimental data on nucleosome repeat lengths.

## Discussion

In this paper, we developed and applied two different methods to investigate the distributions of linker DNA lengths in yeast. Despite being fully independent, and applied to different kinds of experimental data (genomic DNA sequences adjacent to experimentally mapped nucleosomes, and, separately, sequences of biochemically isolated dinucleosomes), both methods lead to the same conclusion: linker DNA lengths are not described by a uniform distribution, but instead are preferentially quantized, obeying the form 10*n*+5 bp.

Our results accord with some, but not others, of the previous experimental studies of linker DNA lengths in yeast. Surprisingly, our Fourier analysis could not detect evidence of periodic higher order structure in the recent genome-wide map of yeast H2A.Z-containing nucleosomes [23], using either their coarse-grained or fine-grained calls. Using a nonredundant subset comprising 1617 of their best-mapped nucleosomes (those which reveal the nucleosome's periodic AA/TT/TA signature [23] our Fourier analysis of dinucleotide frequency in the corresponding extended regions did reveal a ∼10 bp periodicity, with a phase offset *d*_0_ = 5 bp, equivalent to that observed with our smaller number of conventionally sequenced yeast nucleosomes; however this periodicity did not pass a test for significance at the 0.05 level. We suspect that the mapping accuracy of that genome-wide nucleosome collection, which includes nucleosome DNA fragments ranging in length from ∼100–190 bp that are sequenced at only one end, may simply be inadequate to reveal the fine-scale structure revealed by analysis of our conventionally mapped and sequenced nucleosomes.

It is possible that our yeast nucleosome collection may be enriched for an especially stable subset of nucleosomes due to sampling bias imposed by nucleosome mapping technology, and thus could reflect a particular chromatin structure that is enriched in such genome regions. That said, however, our ongoing analysis of more than 50,000 newly mapped unique yeast nucleosome sequences (accounting for ∼67% of the entire genome) leads to exactly the same conclusions regarding linker DNA lengths in yeast (unpublished results), suggesting at least that this linker length form 10*n*+5 is representative of much of the yeast genome.

Nevertheless, we note that our present analysis reveals only a single average most probable linker length distribution. It remains possible that the detailed distribution of linker DNA lengths (and corresponding intrinsic chromatin fiber structures) may vary with location throughout the genome. It is also possible that different species could have different most-probable linker DNA length distributions. Indeed, our ongoing study suggests that linker DNA in human k562 cells human may preferentially occur at lengths that are quantized at 10's. This result however is preliminary and requires further investigation.

Several aspects of our findings are significant. The existence of preferred linker DNA lengths that are constant, modulo the DNA helical repeat, implies an ordered superhelical structure for the average intrinsic chromatin fiber. The spread of detailed linker DNA lengths around the preferred quantized values (Figure 4) could reflect random disorder about an intrinsically ordered structure; or it could actually reflect the *opposite* of that, namely, a tendency to *improve* the local structural order by compensating for inevitable sequence-dependent differences in the intrinsic helical twist of DNA [24]. The 5 bp phase offset means that, on average, consecutive nucleosomes in the yeast genome tend to start from opposite faces of the DNA double helix.

Our work also introduces two approaches for the analysis of linker DNA lengths in any eukaryote for which the needed experimental data are available. In the Fourier analysis, an implicit assumption we made is that the nucleosome cores in the extended regions have the same features as the mapped ones, including the periodicity and relative phases of AA, TT, TA, and GC signals. The justification for this assumption is that these features of nucleosome DNA sequences are thought to reflect the requirement of DNA wrapping in the nucleosome, and to be generic to all nucleosomes [17],[25]. The success of the Fourier method highly depends on both the alignment quality and on the extent to which the linker DNA lengths are actually quantized. A bad alignment tends to degrade the 10 bp periodicity of AA/TT/TA signal in the extended region, just as occurs in the randomly shifted samples (i.e. a randomly shifted sample can be regarded as resulting from randomly aligned nucleosome sequences). In reality the center alignment is not perfect due to various factors such as sequence specificity of the nuclease which is used to biochemically isolate the nucleosomes. Hence we believe that the AA/TT/TA periodicity in the extended region based on a “true” alignment would be even stronger than as obtained in Table 1.

The DHMM provides a general framework for analysis of the linker length distribution function. The components of the DHMM (e.g., the model for the nucleosome sequences or the lengths and sequences of the linker DNA) are not limited to what have been used in this paper: any probabilistic models for the two states can be readily adapted into this framework. The legitimacy of the conclusion regarding the linker DNA length distribution, which is drawn based on the DHMM model, depends on the validity of the model assumptions. Markovian models have proved exceedingly successful in modeling natural DNA or protein sequences in various important problems. In this paper, we proposed a first-order inhomogeneous Markov chain model for the nucleosome state. This model is explicitly designed to characterize the sequential dependence of nucleosomal DNAs in the form of dinucleotides. In addition, it accounts for the variation of signal intensity as a function of positions within the nucleosome region. The need for representing dinucleotides instead of just mononucleotides was explicitly demonstrated in our earlier study [16]. Similarly, the distinction of transition probabilities among positions in the nucleosome region is essential in the prediction of nucleosome positioning, given that the dinucleotide signals are known to be periodic [17],[25]. As expected from these past studies, our training data show that the transition probabilities are NOT homogeneous at different positions across the nucleosome core region. The resulting nucleosome model contains a large number of parameters in the transition matrices (see Methods) because of this time-dependence. Nevertheless, from this perspective, over-fitting is not a big concern in this model. In addition even if this assumption were mis-specified, the trained transition probabilities are still unbiased and consistent estimates of the true parameters. The only loss incurred would be some asymptotic efficiency of the estimates from a statistical point of view. One limitation is that the DHMM is a complex machinery, involving many parameters. Thus we are unable to provide a measure for uncertainty in terms of the entire distribution of linker length, other than the variability around the quantized values quantified by the DHMM-mixture approach. This remains as an open problem.

## Methods

### Data

We obtained 296 nonredundant 142–152 bp long in vivo nucleosome DNA sequences from yeast as described [18]. These sequences were mapped to the genome using BLAST [26]. Dinucleosomes (experimentally isolated chromatin oligomers containing just two nucleosomes) were purified from yeast as described [18], except using less micrococcal nuclease and then gel purifying, cloning, and sequencing protected dinucleosomal DNAs instead of mononucleosomal. We isolated and fully sequenced 335 non-redundant dinucleosomes, with lengths ranging from 280 to 351 bp. These were subsequently filtered (see Results) to yield 214 sequences whose lengths are ≥300 bp. We compared the 296 mononucleosome sequences with the 214 dinucleosome sequences that were at least 300 bp, and found only 4 of them were overlapped. Therefore these two collections can be regarded as two independent sets.

### Fourier Analysis

The center of each experimentally mapped nucleosome DNA sequence was treated as the dyad symmetry axis and was indexed as position 74. We then extended the genomic DNA sequence on both strands in the 3′ direction for 200 bp. The resulting extended sequences were aligned according to the center of the mapped nucleosome sequences (Figures 1A, and 2). We denote the extended sequence as **S** = *s*_1_, …*s_I_*. We sequentially obtained the aligned chunk of DNA of length *L*_0_ from position (147+*d*+1) to (147+*d*+*L*_0_) for *d* = 11, …, 20 bp in the downstream region. *d* is chosen to be greater than 10 bp to avoid sharp peaks observed at the nucleosome boundaries. (Differing values *d* do not influence the observed periodicities, rather, they lead to slight perturbations of amplitude, because of variation of base composition. We then average the results obtained over the set of *d* values.) The average linker DNA length in yeast is ∼20 bp [1]. We therefore chose *L*_0_ = 180 bp such that the extended block roughly covers three full nucleosomes for each sequence. We further generated 500 randomly shifted samples as follows. For each sample, we first generated random shift values *d_i_*∈{11, …, 20} for *i* = 1, … *I*. For each sequence *s_i_*, we extracted the region from position (147+*d_i_*+1) to (147+*d_i_*+*L*_0_). These randomly shifted extended regions were center aligned.

Let *f*(*t*) be the AA/TT/TA frequency (smoothed with a 3 bp moving window to reduce noise from codons) in the *t*th column of the alignment of 296 nucleosome sequences or in the extended regions. We calculated the discrete Fourier transform of *f*(*t*) using *N* = 2000, i.e. 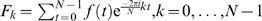. Let 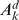 be the amplitude spectrum of *f*(*t*) from the downstream region from position (147+*d*+1) to (147+*d*+*L*_0_) as described in last paragraph. We averaged the amplitude spectrum over *d*'s, i.e. 
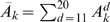
, and plotted *A̅* *_k_* as a function of period in the range 6–20 bp, compared to the amplitude from the original center aligned nucleosomes, and to the average and 95% percentile of the amplitudes from random samples.

### Duration Hidden Markov Model with Kernel Smoothing

For clarity, we first describe our generic duration hidden Markov model (DHMM), which is appropriate for analysis of infinitely long chromatin fibers. We then consider refinements of the model that are necessary for analysis of dinucleosomes.

We model a long chromatin sequence as an oscillating series of two “hidden” states: nucleosome (*N*) and linker (*L*). At the end of each state, the chain must transit to the other state. The nucleosome state has a fixed duration of 147 bp, while the linker state duration (denoted as *d*) has an unknown probability distribution *F_L_*(*d*). We further define probabilistic models for the emission of events within each state. Let **r** = *r*_1_ *r*_2_…*r_m_* be a DNA sequence. Suppose the *N* state has a model *P_N_*(**r**) : = *P*(*r*_1_, …*r_m_*| **r** is in a nucleosome), and the *L* state has *P_L_*(**r**) : = *P*(*r*_1_…*r_m_*| **r** is in a linker) (a subscript “*N*” or “*L*” of *P* is hereafter reserved for the conditional probability given that the sequence is a nucleosome or linker respectively, while a “*P*” without sub-/superscript denotes the probability in general). Note that
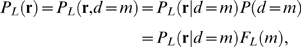
thus the probability distribution of linker length is explicitly modeled here.

For the nucleosome state, we use a first-order time-dependent (inhomogeneous) Markov chain model as in [18], motivated by two facts about nucleosomes: (1) the base composition is sequentially dependent, as revealed by strong patterns of dinucleotide motifs; and (2) the pattern varies as a function of position in the nucleosome (referred to as time here) [16],[17]. A time-dependent Markov chain captures the sequential dependence while allowing heterogeneity across different positions. More explicitly, let 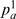 be the probability of observing the letter “*a*” at position 1 (*a* = A, C, G or T); and let 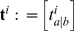 be a 4×4 transition matrix specifying probabilities of observing *a* at position (*i*+1) given *b* at position *i* for *i* = 1, …, 146. Then for any given nucleosome sequence = *e*_1_…*e*_147_,
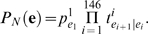
We model the linker state with a homogeneous Markov chain, which can be fully defined by the initial base composition denoted as *q_e_* for (*e* = *A*,*C*,*G*,*T*) and a single transition matrix **v** : = [*v_a_*_|*b*_] (defined analogously to **t** *^i^* above). For any linker DNA sequence e = *e*_1_…*e_m_*,
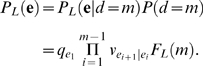



For a DNA sequence **r** = *r*_1_ *r*_2_…*r_m_* (Watson strand) and its reverse complementary (Crick) strand 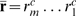, let *π* = *π*_0_ *π*_1_…*π_k_*…*π_K_π_K_*_+1_ be the path of underlying hidden states. The states *π*_0_,*π_K_*_+1_ are the initial and ending states without emission (“silent” states). The state *π_k_*, equal to *N* or *L*, is associated with a duration *d_k_*, such that 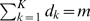 (if *π_k_* = *N*, then *d_k_* = 147). Let the probability that a random sequence starts with the *N* state be *τ*, and that of ending in *N* be *γ* (i.e., *P*(*π*_1_ = *N*|*π*_0_) = τ, *P*(*π_K_*_+1_|*π_K_* = *N*) = *γ*). We set *γ* equal to *τ* throughout this paper, assuming balanced digestion at the two ends of the dinucleosomes during their biochemical isolation. Now suppose that π partitions **r** into *m*_1_ nucleosomes on the Watson strand: 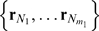, and *m*_2_ interwoven linkers: 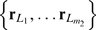, corresponding to 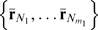 and 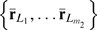 on the Crick strand (note: |*m*_1_−*m*_2_|≤1). Then
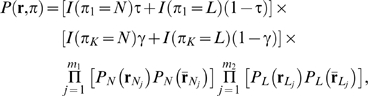
(1)where *I*()is an indicator function. The exact value of *m*_1_ or *m*_2_ depends on the length of DNA sequence under modeling. For the dinucleosome data, the value for *m*_1_ is restricted to 2, while the value for *m*_2_ can be 1, 2, or 3. Using dynamic programming (e.g., [27]), one can find the optimal path π^*^ that maximizes the probability, i.e.
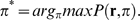
(2)


For dinucleosomes, the standard DHMM needs to be modified to reflect that the first and last non-silent states, i.e., π_1_ and π*_K_*, are in general truncated due to the extensive nuclease digestion used to maximize the yield of these short chromatin fragments. In other words, the duration for *N* and *L* at π_1_ and π*_K_* are different from internal π*_K_*'s. Let 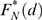 and 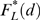 be the duration distribution of *N* and *L* states at π_1_ or π*_K_*. If π_1_ = *N* and the corresponding emission is 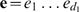 (*d*_1_≤147), then 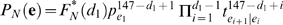, where 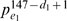 is the occurrence probability of letter *e*_1_ at nucleosome position 147−*d*_1_+1. If π_1_ = *L*, then *P_L_*(**e**) must be calculated using 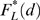 in equation (1). Analogous modifications apply to π*_K_*.

Based on the center alignment of the 296 mononucleosome sequences, we trained a nucleosome model as follows. The probability for letter *e* at position *j*, i.e. 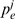 (*e* = *A*/*C*/*G*/*T*), was estimated as the fraction of *e* at the *j*th column of the alignment of both strands for *j* = 1,…147. Likewise the transition probability 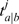 in **t** *^j^* was estimated as the fraction of occurrence *a* at position *j*+1 given *b* at position *j*. The transition probabilities for the nucleosome model were again smoothed using a 3 bp moving window.

**Figure 7:**
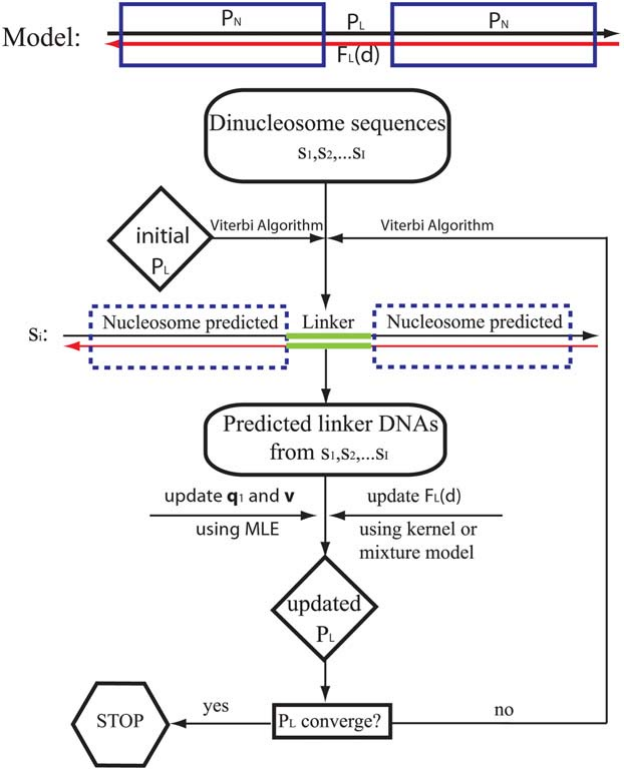
A diagram for the DHMM and linker DNA length estimation procedure. The DHMM contains two oscillating states: nucleosome (*N*) and linker (*L*). The nucleosome state model *P_N_* is defined as a heterogeneous Markov chain trained based on the nucleosome sequence alignment, and is fixed throughout the DHMM. The linker state model (*P_L_*) is a homogeneous Markov chain defined by base composition at the first position q_1_, the transition matrix v and the linker length distribution (duration) *F_L_*(*d*). The linker model is updated iteratively until convergence using the predicted linker DNAs between two nucleosomes. In particular, the linker length distribution *F_L_*(*d*) is estimated using a kernel smoothing method or a mixture model method.

Let *s_i_*, *i* = 1,…*I* be the dinucleosome sequences. We set 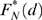 to be a uniform distribution on [147−*α*, …, 147] and 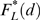 to be a uniform distribution on [1, 2, …, *β*], where *α* measures the maximum possible nucleosome truncation (i.e. maximal over-digestion into a nucleosome at either end of the dinucleosome) and *β* the maximum extra linker DNA length at either end of the dinucleosome. The ideal values of *α* and *β* should be chosen according to the real extent of truncation or extra linker DNA at two ends in the population of dinucleosomes, as isolated biochemically. If *α* or *β* is set too small, systematic biases in the resulting linker length distribution will result, as the path π predicted by the model must satisfy these constraints. On the other hand, choosing over-large values of *α* and *β* will inflate path space, degrading the precision of the predictions. We chose *α* and *β* to be 20 and 30 bp, respectively. The linker length distribution is initialized as uniform in [1, …, *c*], where *c* = 50. The linker DNA initial base composition (*q_e_*) and the transition matrix **v** are initialized equal to the corresponding average probabilities from the mononucleosome data. The linker length distribution is estimated iteratively, as follows (see the flow chart in Figure 7):

1. Predict the optimal path π*_i_* given the current parameter estimates for each sequence *s_i_*, *i* = 1,…*I*.
2. Update the linker length distribution using the kernel smoothing method (see below) based on the length of predicted linker between the two putative nucleosomes.
3. Update the linker base composition *q_e_* for *e* = *A*/*C*/*G*/*T* and transition probability matrix **v** based on the predicted internal linkers.
4. Repeat step 1, 2, 3 until the linker length distribution converges.

The empirical distribution of *d* from step 1 is noisy prior to convergence, therefore we employed a standard kernel smoothing technique with bandwidth of 1.5 bp to improve the estimation under a Gaussian kernel [28].

### DHMM with Mixture Distribution

Our results using the DHMM kernel smoothing method suggest that linker DNAs preferentially occur according to the form 10*n*+*ε* where *ε* is a random term whose density has mode at a constant *d*_0_, such that 0≤*d*_0_<10, but with variability, i.e., that *ε* can take values *d*_0_, *d*_0_±1, ±2 etc.. If this model holds, Fig. 4 suggests that *d*_0_≈5. In the extreme case, where Var(ε) = 0, the linker lengths would be strictly quantized with the form 10*n*+*d*_0_.

We characterize such a distribution with a *location mixture model*. Let *μ* = (*μ*_1_,…,*μ_K_*) be the peak positions in a projected range [1,2,…*c*], where each *μ_k_* indexes a *location* distribution with density *f*(*d*;*μ_k_*), i.e.: *f*(*d*;*μ_k_*)≡*f*(*d*−*μ_k_*) for *k* = 1,…*K*. The linker length value around *μ_k_* is locally distributed as *f*(*d*;*μ_k_*). Suppose that the probability of observing a linker length *d* from the *k*th component is η*_k_* (and hence 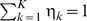). Let η = (η_1_,…,η*_K_*). Then the marginal distribution of *d* can be written as 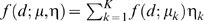.

Based on the results of the Fourier and DHMM-kernel smoothing analyses, we impose the constraint that these components are equally spaced with 10 bp period, i.e. *μ_k_* = *μ*_1_+10*(*k*−1) for *k* = 1,…*K*. Under this model, the between-component distance is fixed throughout the algorithm. The absolute positions of components *μ*_1_,…*μ_K_* are varied as a whole to maximize the likelihood as the weights η are simultaneously updated.

We modeled *f* with a Gaussian mixture distribution with a common standard deviation σ. The Gaussian density is normalized since the linker length distribution is discrete. The initial value of *μ_k_* was set as 10*k+2.5 or 10*(*k*−1)+7.5, with equal weight 1/*K* for each *k* = 1,…*K*, and with *K* = 5. (We confirmed that the peak positions resulting from this analysis are consistent under different starting values). We follow the same four steps as in the kernel smoothing method, except that step 2 is replaced by a standard expectation-maximization algorithm (EM, [29]), in which (*μ*,η,σ) are updated using predicted linker lengths.

### Availability

The mono- and dinucleosome sequences and some codes used in the paper will be available at http://bioinfo.stats.northwestern.edu/jzwang.

## Supporting Information

Figure S1 Linker DNA length distribution (*F_L_*(*d*)) for random (A) or shuffled (B) dinucleosome DNA sequences, using the DHMM-kernel smoothing method. (A) Random dinucleosome sequences simulated based on the product multinomial model with the same base composition and the same length distribution as in the real data. The DHMM-kernel procedure was followed exactly as in Methods. In both simulations, the resulting linker length distribution varied between trials, and the ∼10 bp periodicity disappeared in general. (B) As in (A) except using sequences obtained by shuffling true dinucleosome sequences while keeping the dinucleotide frequency fixed.
(0.41 MB EPS)

## References

[1] van Holde KE (1989). Chromatin.

[2] Luger K, Richmond T (1998). DNA binding within the nucleosome core.. Curr Opin Struct Biol.

[3] Schalch T, Duda S, Sargent DF, Richmond T (2005). X-ray structure of a tetranucleosome and its implications for the chromatin fibre.. Nature.

[4] Robinson PJ, Fairall L, Huynh VA, Rhodes D (2006). EM measurements define the dimensions of the “30-nm” chromatin fiber: evidence for a compact, interdigitated structure.. Proc Natl Acad Sci U S A.

[5] Lohr D, van Holde KE (1979). Organization of spacer DNA in chromatin.. Proc Natl Acad Sci U S A.

[6] Strauss F, Prunell A (1983). Organization of internucleosomal DNA in rat liver chromatin.. EMBO J.

[7] Ulanovsky LE, Trifonov EN (1985). A different view point on the chromatin higher order structure: steric exclusion effects..

[8] Widom J (1992). A relationship between the helical twist of DNA and the ordered positioning of nucleosomes in all eukaryotic cells.. Proc Natl Acad Sci U S A.

[9] Bertin A, Leforestier A, Durand D, Livolant F (2004). Role of histone tails in the conformation and interactions of nucleosome core particles.. Biochemistry.

[10] Cui Y, Bustamante C (2000). Pulling a single chromatin fiber reveals the forces that maintain its higher-order structure.. Proc Natl Acad Sci U S A.

[11] Cohanim AB, Kashi Y, Trifonov EN (2005). Yeast nucleosome DNA pattern: deconvolution from genome sequences of S. cerevisiae.. J Biomol Struct Dyn.

[12] Cohanim AB, Kashi Y, Trifonov EN (2006). Three sequence rules for chromatin.. J Biomol Struct Dyn.

[13] Kato M, Onishi Y, Wada-Kiyama Y, Abe T, Ikemura T (2003). Dinucleosome DNA of human k562 cells: experimental and computational characterizations.. J Mol Biol.

[14] Strauss F, Prunell A (1982). Nucleosome spacing in rat liver chromatin. a study with exonuclease III.. Nucleic Acids Res.

[15] Satchwell S, Travers A (1989). Asymmetry and polarity of nucleosomes in chicken erythrocyte chromatin.. EMBO J.

[16] Wang JPZ, Widom J (2005). Improved alignment of nucleosome DNA sequences using a mixture model.. Nucleic Acids Research.

[17] Satchwell S, Drew H, Travers A (1986). Sequence periodicities in chicken nucleosome core DNA.. J Mol Biol.

[18] Segal E, Fondufe-Mittendorf Y, Chen L, Thåström A, Field Y (2006). A genomic code for nucleosome positioning.. Nature.

[19] Hörz W, Altenburger W (1981). Sequence specific cleavage of DNA by micrococcal nuclease.. Nucleic Acids Res.

[20] Yuan GC, Liu YJ, Dion MF, Slack MD, Wu LF (2005). Genome-scale identification of nucleosome positions in S. cerevisiae.. Science.

[21] Rabiner LR (1989). A tutorial on hidden markov models and selected applications in speech recognition.. Proc IEEE.

[22] Thomas J, Furber V (1976). Yeast chromatin structure.. FEBS Lett.

[23] Albert I, Mavrich TN, Tomsho LP, Qi J, Zanton SJ (2007). Translational and rotational settings of H2A.Z nucleosomes across the Saccharomyces cerevisiae genome.. Nature.

[24] Olson WK, Gorin AA, Lu XJ, Hock LM, Zhurkin VB (1998). DNA sequence-dependent deformability deduced from protein–DNA crystal complexes.. Proc Natl Acad Sci U S A.

[25] Widom J (2001). Role of DNA sequence in nucleosome stability and dynamics.. Q Rev Biophys.

[26] Altschul SF, Gish W, Miller W, Myers EW, Lipman DJ (1990). Basic local alignment search tool.. J Mol Biol.

[27] Durbin R, Eddy SR, Krogh A, Mitchison G (1998). Biological Sequence Analysis: Probabilistic Models of Proteins and Nucleic Acids.

[28] Silverman BW (1992). Density Estimation for Statistics and Data Analysis.

[29] Dempster A, Laird N, Rubin D (1977). Maximum likelihood from incomplete data via the EM algorithm.. J R Stat Soc Ser B.

